# 1-[4-({4-[(*E*)-(2-Hy­droxy­naphthalen-1-yl)methyl­idene­amino]­phen­yl}sulfan­yl)phen­yl]ethanone

**DOI:** 10.1107/S1600536812049835

**Published:** 2012-12-12

**Authors:** Rabihe Hebbachi, Hénia Mousser, Abdelhamid Mousser

**Affiliations:** a Département de Chimie, Faculté des Sciences Exactes, Université Mentouri Constantine, Route de Ain El Bey, Constantine, Algerie; b Département de Chimie Industrielle, Faculté des Sciences de l’Ingénieur, Université Mentouri Constantine, Campus Chaab Erssas, Constantine, Algerie

## Abstract

The title Schiff base compound, C_25_H_19_NO_2_S, crystallizes in a statistically disordered structure comprising keto and enol tautomeric forms. In the enol form, the benzenoid arrangment is promoted by a strong intra­molecular O—H⋯N hydrogen bond and adopts an *E* conformation about the imine bond. In the keto form there is an intramolecular N—H⋯O hydrogen bond. In the crystal, an extended network of C—H⋯O hydrogen bonds stabilizes columns parallel to the *c* axis, forming large voids (there are four cavities of 108 Å^3^ per unit cell) with highly disordered residual electron density. The SQUEEZE procedure in *PLATON* [Spek (2009 ▶). *Acta Cryst.* D**65**, 148–155] was used to eliminate the contribution of this electron density from the intensity data, and the solvent-free model was employed for the final refinement. The contribution of this undetermined solvent was ignored in the calculation of the unit-cell characteristics.

## Related literature

For related structures, see: Blagus & Kaitner (2011 ▶); Farag *et al.* (2010 ▶); Venkatachalam *et al.* (2011 ▶). For background to Schiff bases and their applications, see: Li *et al.* (2003 ▶); Villar *et al.* (2004 ▶); Kagkelari *et al.* (2009 ▶); Ourari *et al.* (2008 ▶); Zidane *et al.* (2011 ▶).
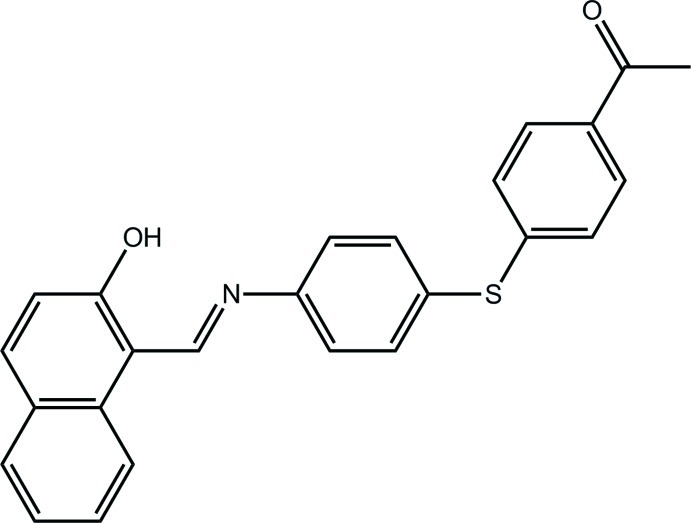


## Experimental

### 

#### Crystal data

C_25_H_19_NO_2_S
*M* *_r_* = 397.47
Monoclinic, 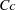

*a* = 10.695 (3) Å
*b* = 44.458 (14) Å
*c* = 4.4437 (11) Å
β = 99.004 (9)°
*V* = 2086.8 (10) Å^3^
*Z* = 4
Mo *K*α radiation
μ = 0.18 mm^−1^
*T* = 150 K
0.58 × 0.17 × 0.06 mm

#### Data collection

Bruker APEXII diffractometer
Absorption correction: multi-scan (*SADABS*; Sheldrick, 2002 ▶) *T*_min_ = 0.898, *T*_max_ = 0.990
8026 measured reflections
3680 independent reflections
2952 reflections with *I* > 2σ(*I*)
*R*_int_ = 0.036

#### Refinement

*R*[*F* ^2^ > 2σ(*F* ^2^)] = 0.046
*wR*(*F* ^2^) = 0.125
*S* = 0.98
3680 reflections
263 parameters
2 restraints
H-atom parameters constrained
Δρ_max_ = 0.24 e Å^−3^
Δρ_min_ = −0.22 e Å^−3^
Absolute structure: Flack (1983 ▶), 1291 Friedel pairs
Flack parameter: −0.06 (10)

### 

Data collection: *APEX2* (Bruker, 2006 ▶); cell refinement: *SAINT* (Bruker, 2006 ▶); data reduction: *SAINT*; program(s) used to solve structure: *SIR97* (Altomare *et al.*, 1999 ▶); program(s) used to refine structure: *SHELXL97* (Sheldrick, 2008 ▶); molecular graphics: *ORTEP-3 for Windows* (Farrugia, 2012 ▶); software used to prepare material for publication: *WinGX* (Farrugia, 2012 ▶).

## Supplementary Material

Crystal structure: contains datablock(s) global, I. DOI: 10.1107/S1600536812049835/tk5178sup1.cif

Structure factors: contains datablock(s) I. DOI: 10.1107/S1600536812049835/tk5178Isup2.hkl

Supplementary material file. DOI: 10.1107/S1600536812049835/tk5178Isup3.cml

Additional supplementary materials:  crystallographic information; 3D view; checkCIF report

## Figures and Tables

**Table 1:** Hydrogen-bond geometry (Å, °)

*D*—H⋯*A*	*D*—H	H⋯*A*	*D*⋯*A*	*D*—H⋯*A*
O1*A*—H1*A*⋯N13*A*	0.84	1.80	2.558 (4)	149
N13*B*—H13*B*⋯O1*B*	0.88	1.85	2.558 (4)	136
C9—H9⋯O28^i^	0.95	2.46 (1)	3.398 (4)	168
C19*A*—H19*A*⋯O28^i^	0.95	2.56 (1)	3.506 (4)	174
C22—H22⋯O1*A* ^ii^	0.95	2.44 (1)	3.337 (4)	157
C27—H27*B*⋯O1*A* ^i^	0.98	2.49 (1)	3.442 (4)	164

Symmetry codes: (i) 
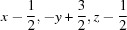
; (ii) 
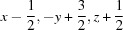
.

**Fig. 1.**
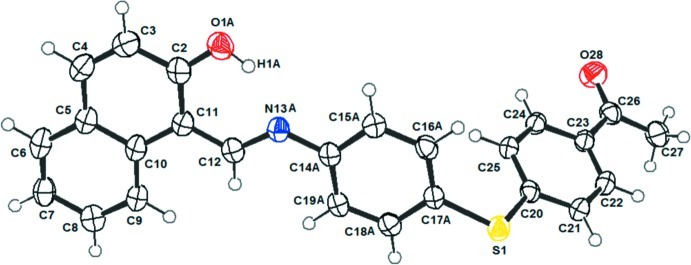
The molecular structure of (I) with atom labels and 50% probability displacement ellipsoids for non-H atoms. Disorder is present between the (illustrated) enol and keto forms.

**Fig. 2.**
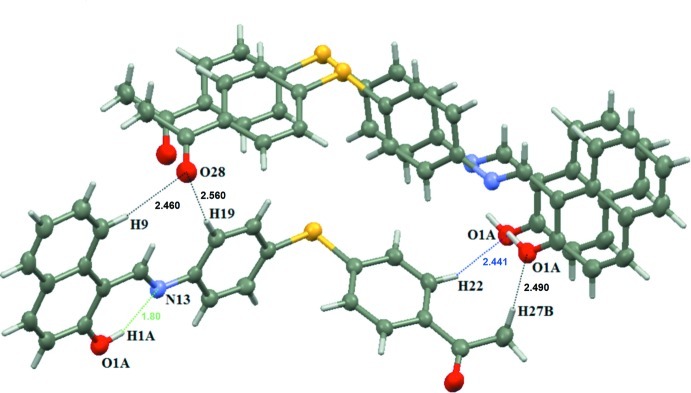
The four intermolecular C—H···O interactions bonds (symmetry codes: C9—H9···O28i, C19A—H19A···O28i, C27—H27B···O1Ai [(i): x - 1/2, -y + 1/2, z - 1/2] and C22–H22···O1Aii [(ii): x - 1/2, -y + 1/2, z + 1/2]).

**Fig. 3.**
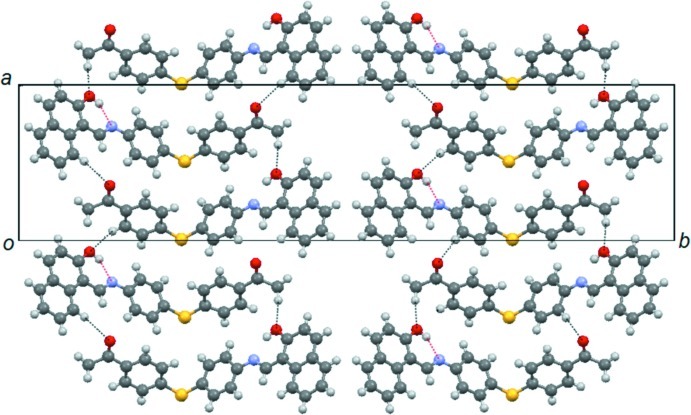
A view of the unit-cell contents in projection down the c axis in (I), highlighting large void channels within the unit cell.

## supplementary crystallographic information

### Comment

Schiff bases are important compounds owing to their wide range of biological activities and industrial applications (Li *et al.*, 2003; Villar *et al.*, 2004). They have also been used as ligands in coordination chemistry (Kagkelari *et al.*, 2009; Ourari *et al.* 2008; Zidane *et al.*, 2011). Schiff bases are generally synthesized by nucleophilic condensation of an aromatic amine and a carbonyl compound, followed by the dehydration of the hemiaminal intermediate to generate the imine (Blagus *et al.*, 2011).

In the present paper, we describe the synthesis and structural study of *E*-2-{[4-(4-acetylphenylsulfanyl)phenyl-amino]methyl} 2-oxo-naphthalene. The titled compound (Fig. 1) crystallizes in a disordered keto–amino tautomer [C*sp*^2^—O 1.277 (4) Å]. The C12—N13A bond length [1.334 (4) Å] is longer than C═N but in the same range of those observed in the literature for related compounds (Blagus *et al.*, 2011; Farag *et al.*, 2010; Venkatachalam *et al.*, 2011) in accordance with the observed keto–amino tautomer form. The benzenoid arrangement is promoted by a strong intramolecular hydrogen bond O—H···N [N···O 2.558 (4) Å].

The Schiff base adopts a *E* conformation about the C12═N13 bond with a C11—C12—N13A—C14A torsion angle = -178.5 (3) Å. The central part of the molecule is planar with a dihedral angle between the benzene and naphthalene rings being less than 1 °. The molecule is twisted around the sulfide atom, so the average dihedral angle between the acetyl phenyl ring and the oxo naphthalen ring system is about 71°. The electron delocalisation between the two sulfur-bound lone pairs and π electrons of the adjacent phenyl rings leads to a slightly tighter S*sp*^3^ angle (C17A—S1—C20 = 104.88 (15)°). The two similar sulfide carbone single bonds [C17A—S1 1.780 (3) Å and C20—S1 1.764 (3) Å] are as expected. The short bond C3—C4 distance [1.354 (5) Å] adjacent to the O1 oxygen atom of the naphthalen core indicates the presence of quinoid effect.

In the crystal, molecules are aligned head to foot along *b* axis, in columns parallel to [0 0 1] axis and the structure is stabilized by four kinds of C—H···O interactions (Fig. 2, Table 1). This arrangement separates the equivalent groups in columns by 4.444 (1) Å.

The large void channels in the structure (Fig. 3) contains residual electrons density with high disorder. The residual electron density were difficult to model and therefore, the SQUEEZE function of *PLATON* (Spek, 2009) was used to eliminate the contribution of the electron density in the solvent region from the intensity data, and the solvent-free model was employed for the final refinement. There are four cavities of 108 Å^3^ per unit cell. *PLATON* estimated that each cavity contains 12 electrons which may correspond to a solvent molecule.

### Experimental

The title Schiff base was prepared by the condensation of 4-amino-4-acetyl diphenylsulfide and 2-hydroxy naphthaldehyde in a 1:1 molar ratio in ethanol solution. The mixture was stirred under reflux three hours. The crystals of title compound crystallized from a mixture of chloroforme/hexane (1/1). The orange needles were collected by filtration and dried in air. Yield: 61%. Melting Point: 451 K.

### Refinement

H atoms were positioned geometrically, using a riding model with C—H = 0.98 Å [*U*_iso_(H) = 1.5 × *U*_eq_(methyl-C)] and with C—H = 0.95 Å [*U*_iso_(H) =1.2 × *U*_eq_(aromatic-C)]. The model included free rotation about the C—C(methyl) bond

Since one hydrogen is not very well localized between N13 and O1, the structure is described as the presence of two tautomers. Hydrogen is bonded to O1 in the first one (part A) and to N13 in the second (part B). This disorder was modeled by refining part A (except H on N13), with O—H = 0.84 Å (*U*_iso_(H) = 1.5), and part B (except H on O1) with equivalence of N13 and central phenyl ring (C14 to C19) atoms, with N—H = 0.88 Å (*U*_iso_(H) = 1.5).

Large voids in the structure contains residual electrons density with hight disorder and or thermal motions. The SQUEEZE procedure of *PLATON* was used to eliminate the contribution of this residual electron density from the intensity data, and the solvent-free model was employed for the final refinement.

### Crystal data

**Table:**

C_25_H_19_NO_2_S	*F*(000) = 832
*M**_r_* = 397.47	*D*_x_ = 1.265 Mg m^−^^3^
Monoclinic, *C**c*	Mo *K*α radiation, λ = 0.71073 Å
Hall symbol: C -2yc	Cell parameters from 1817 reflections
*a* = 10.695 (3) Å	θ = 2.4–26.0°
*b* = 44.458 (14) Å	µ = 0.18 mm^−^^1^
*c* = 4.4437 (11) Å	*T* = 150 K
β = 99.004 (9)°	Stick, orange
*V* = 2086.8 (10) Å^3^	0.58 × 0.17 × 0.06 mm
*Z* = 4	

### Data collection

**Table:**

Bruker APEXII diffractometer	2952 reflections with *I* > 2σ(*I*)
Graphite monochromator	*R*_int_ = 0.036
CCD rotation images, thin slices scans	θ_max_ = 27.5°, θ_min_ = 3.0°
Absorption correction: multi-scan (*SADABS*, Sheldrick, 2002)	*h* = −12→13
*T*_min_ = 0.898, *T*_max_ = 0.990	*k* = −57→57
8026 measured reflections	*l* = −4→5
3680 independent reflections	

### Refinement

**Table:**

Refinement on *F*^2^	Secondary atom site location: difference Fourier map
Least-squares matrix: full	Hydrogen site location: inferred from neighbouring sites
*R*[*F*^2^ > 2σ(*F*^2^)] = 0.046	H-atom parameters constrained
*wR*(*F*^2^) = 0.125	*w* = 1/[σ^2^(*F*_o_^2^) + (0.0718*P*)^2^] where *P* = (*F*_o_^2^ + 2*F*_c_^2^)/3
*S* = 0.98	(Δ/σ)_max_ = 0.005
3680 reflections	Δρ_max_ = 0.24 e Å^−^^3^
263 parameters	Δρ_min_ = −0.22 e Å^−^^3^
2 restraints	Absolute structure: Flack (1983), 1291 Friedel pairs
Primary atom site location: structure-invariant direct methods	Flack parameter: −0.06 (10)

### Special details

**Table:**

Geometry. All e.s.d.'s (except the e.s.d. in the dihedral angle between two l.s. planes) are estimated using the full covariance matrix. The cell e.s.d.'s are taken into account individually in the estimation of e.s.d.'s in distances, angles and torsion angles; correlations between e.s.d.'s in cell parameters are only used when they are defined by crystal symmetry. An approximate (isotropic) treatment of cell e.s.d.'s is used for estimating e.s.d.'s involving l.s. planes.
Refinement. Refinement of *F*^2^ against ALL reflections. The weighted *R*-factor *wR* and goodness of fit *S* are based on *F*^2^, conventional *R*-factors *R* are based on *F*, with *F* set to zero for negative *F*^2^. The threshold expression of *F*^2^ > σ(*F*^2^) is used only for calculating *R*-factors(gt) *etc*. and is not relevant to the choice of reflections for refinement. *R*-factors based on *F*^2^ are statistically about twice as large as those based on *F*, and *R*- factors based on ALL data will be even larger.Since hydrogen is not very well localized between N13 and O1, the structure is described as the presence of two tautomers. Hydrogen is bonded to O1 in the first one (part A) and to N13 in the second (part B). This disorder was modeled by refining part A (except H on N13) and part B (except H on O1) with equivalence of N13 and central phenyl ring (C14 to C19) atoms.

### Fractional atomic coordinates and isotropic or equivalent isotropic displacement parameters (Å^2^)

**Table:**

	*x*	*y*	*z*	*U*_iso_*/*U*_eq_	Occ. (<1)
O1A	0.9261 (2)	0.89274 (5)	0.2227 (5)	0.0432 (6)	0.5
H1A	0.8804	0.8781	0.2548	0.065*	0.5
O1B	0.9261 (2)	0.89274 (5)	0.2227 (5)	0.0432 (6)	0.5
C2	0.8665 (3)	0.90927 (7)	0.0128 (7)	0.0343 (7)	
C3	0.9267 (3)	0.93567 (7)	−0.0798 (8)	0.0404 (8)	
H3	1.0096	0.9405	0.0187	0.048*	
C4	0.8695 (3)	0.95396 (7)	−0.3033 (8)	0.0404 (8)	
H4	0.9135	0.9711	−0.3594	0.048*	
C5	0.7436 (3)	0.94802 (7)	−0.4576 (7)	0.0339 (7)	
C6	0.6843 (4)	0.96779 (7)	−0.6875 (8)	0.0402 (8)	
H6	0.7291	0.9849	−0.7408	0.048*	
C7	0.5643 (3)	0.96264 (7)	−0.8334 (7)	0.0423 (9)	
H7	0.5253	0.9761	−0.9862	0.051*	
C8	0.4992 (4)	0.93702 (8)	−0.7531 (7)	0.0408 (8)	
H8	0.4155	0.9332	−0.853	0.049*	
C9	0.5551 (3)	0.91739 (7)	−0.5316 (8)	0.0357 (7)	
H9	0.5092	0.9003	−0.4825	0.043*	
C10	0.6785 (3)	0.92219 (6)	−0.3773 (7)	0.0317 (7)	
C11	0.7411 (3)	0.90245 (6)	−0.1401 (7)	0.0306 (7)	
C12	0.6792 (3)	0.87685 (7)	−0.0513 (7)	0.0321 (7)	
H12	0.5963	0.8725	−0.153	0.039*	
N13A	0.7319 (3)	0.85845 (5)	0.1698 (6)	0.0320 (6)	0.5
C14A	0.6753 (3)	0.83248 (6)	0.2696 (7)	0.0285 (6)	0.5
C15A	0.7471 (3)	0.81538 (7)	0.4960 (7)	0.0325 (7)	0.5
H15A	0.8306	0.8216	0.5776	0.039*	0.5
C16A	0.6980 (3)	0.78934 (7)	0.6035 (7)	0.0343 (7)	0.5
H16A	0.7467	0.7782	0.7626	0.041*	0.5
C17A	0.5781 (3)	0.77958 (6)	0.4798 (7)	0.0296 (7)	0.5
C18A	0.5070 (3)	0.79663 (7)	0.2544 (7)	0.0324 (7)	0.5
H18A	0.4242	0.7901	0.1706	0.039*	0.5
C19A	0.5541 (3)	0.82303 (7)	0.1483 (7)	0.0345 (7)	0.5
H19A	0.504	0.8345	−0.0058	0.041*	0.5
N13B	0.7319 (3)	0.85845 (5)	0.1698 (6)	0.0320 (6)	0.5
H13B	0.8087	0.8629	0.2611	0.038*	0.5
C14B	0.6753 (3)	0.83248 (6)	0.2696 (7)	0.0285 (6)	0.5
C15B	0.7471 (3)	0.81538 (7)	0.4960 (7)	0.0325 (7)	0.5
H15B	0.8306	0.8216	0.5776	0.039*	0.5
C16B	0.6980 (3)	0.78934 (7)	0.6035 (7)	0.0343 (7)	0.5
H16B	0.7467	0.7782	0.7626	0.041*	0.5
C17B	0.5781 (3)	0.77958 (6)	0.4798 (7)	0.0296 (7)	0.5
C18B	0.5070 (3)	0.79663 (7)	0.2544 (7)	0.0324 (7)	0.5
H18B	0.4242	0.7901	0.1706	0.039*	0.5
C19B	0.5541 (3)	0.82303 (7)	0.1483 (7)	0.0345 (7)	0.5
H19B	0.504	0.8345	−0.0058	0.041*	0.5
S1	0.50675 (10)	0.747532 (16)	0.62296 (19)	0.0362 (2)	
C20	0.5814 (3)	0.71639 (6)	0.4809 (7)	0.0288 (7)	
C21	0.5376 (3)	0.68797 (7)	0.5525 (7)	0.0342 (7)	
H21	0.47	0.6864	0.6674	0.041*	
C22	0.5921 (3)	0.66202 (7)	0.4567 (7)	0.0344 (7)	
H22	0.5608	0.6429	0.5051	0.041*	
C23	0.6915 (3)	0.66362 (7)	0.2916 (7)	0.0309 (7)	
C24	0.7347 (3)	0.69227 (7)	0.2182 (7)	0.0317 (7)	
H24	0.8024	0.6938	0.1038	0.038*	
C25	0.6800 (3)	0.71807 (7)	0.3103 (7)	0.0305 (7)	
H25	0.7098	0.7372	0.257	0.037*	
C26	0.7554 (3)	0.63638 (7)	0.1932 (8)	0.0367 (8)	
C27	0.7015 (4)	0.60589 (7)	0.2401 (9)	0.0474 (9)	
H27A	0.7546	0.5904	0.1671	0.071*	
H27B	0.6154	0.6046	0.1264	0.071*	
H27C	0.6992	0.6028	0.4576	0.071*	
O28	0.8519 (3)	0.63891 (6)	0.0759 (6)	0.0530 (7)	

### Atomic displacement parameters (Å^2^)

**Table:**

	*U*^11^	*U*^22^	*U*^33^	*U*^12^	*U*^13^	*U*^23^
O1A	0.0444 (14)	0.0349 (12)	0.0494 (16)	0.0022 (10)	0.0045 (11)	−0.0044 (10)
O1B	0.0444 (14)	0.0349 (12)	0.0494 (16)	0.0022 (10)	0.0045 (11)	−0.0044 (10)
C2	0.044 (2)	0.0286 (16)	0.032 (2)	0.0051 (14)	0.0121 (14)	−0.0093 (13)
C3	0.0406 (19)	0.0355 (17)	0.047 (2)	−0.0029 (15)	0.0120 (16)	−0.0151 (15)
C4	0.048 (2)	0.0319 (16)	0.046 (2)	−0.0078 (15)	0.0241 (17)	−0.0102 (15)
C5	0.048 (2)	0.0278 (15)	0.0299 (19)	−0.0021 (14)	0.0177 (14)	−0.0072 (12)
C6	0.064 (2)	0.0293 (16)	0.0320 (19)	−0.0051 (15)	0.0218 (16)	−0.0041 (13)
C7	0.061 (2)	0.0339 (17)	0.033 (2)	−0.0001 (16)	0.0109 (17)	0.0036 (14)
C8	0.049 (2)	0.0396 (18)	0.034 (2)	0.0013 (15)	0.0062 (16)	−0.0023 (14)
C9	0.048 (2)	0.0294 (15)	0.0310 (18)	−0.0037 (14)	0.0092 (14)	−0.0008 (13)
C10	0.0457 (19)	0.0240 (13)	0.0287 (18)	−0.0010 (13)	0.0159 (14)	−0.0066 (12)
C11	0.0410 (18)	0.0249 (14)	0.0279 (17)	−0.0011 (13)	0.0112 (13)	−0.0068 (12)
C12	0.0397 (18)	0.0296 (15)	0.0279 (18)	0.0039 (13)	0.0082 (13)	−0.0055 (12)
N13A	0.0375 (14)	0.0286 (13)	0.0308 (15)	0.0022 (11)	0.0082 (11)	−0.0019 (11)
C14A	0.0362 (17)	0.0263 (14)	0.0248 (17)	0.0041 (12)	0.0101 (12)	−0.0052 (12)
C15A	0.0359 (18)	0.0332 (16)	0.0278 (19)	0.0001 (13)	0.0034 (13)	−0.0021 (12)
C16A	0.0368 (18)	0.0370 (17)	0.0284 (19)	0.0042 (14)	0.0029 (14)	0.0051 (13)
C17A	0.0358 (18)	0.0267 (14)	0.0270 (18)	0.0034 (12)	0.0069 (13)	0.0017 (12)
C18A	0.0342 (17)	0.0327 (16)	0.0312 (19)	0.0045 (13)	0.0078 (13)	−0.0033 (12)
C19A	0.0403 (18)	0.0311 (16)	0.0311 (19)	0.0048 (13)	0.0030 (14)	0.0036 (13)
N13B	0.0375 (14)	0.0286 (13)	0.0308 (15)	0.0022 (11)	0.0082 (11)	−0.0019 (11)
C14B	0.0362 (17)	0.0263 (14)	0.0248 (17)	0.0041 (12)	0.0101 (12)	−0.0052 (12)
C15B	0.0359 (18)	0.0332 (16)	0.0278 (19)	0.0001 (13)	0.0034 (13)	−0.0021 (12)
C16B	0.0368 (18)	0.0370 (17)	0.0284 (19)	0.0042 (14)	0.0029 (14)	0.0051 (13)
C17B	0.0358 (18)	0.0267 (14)	0.0270 (18)	0.0034 (12)	0.0069 (13)	0.0017 (12)
C18B	0.0342 (17)	0.0327 (16)	0.0312 (19)	0.0045 (13)	0.0078 (13)	−0.0033 (12)
C19B	0.0403 (18)	0.0311 (16)	0.0311 (19)	0.0048 (13)	0.0030 (14)	0.0036 (13)
S1	0.0411 (4)	0.0342 (4)	0.0357 (5)	0.0028 (4)	0.0138 (3)	0.0048 (4)
C20	0.0311 (16)	0.0305 (15)	0.0233 (18)	0.0001 (12)	−0.0005 (12)	0.0028 (12)
C21	0.0350 (18)	0.0354 (17)	0.033 (2)	−0.0023 (13)	0.0092 (13)	0.0063 (13)
C22	0.0394 (18)	0.0310 (16)	0.0326 (19)	−0.0070 (13)	0.0047 (14)	0.0037 (13)
C23	0.0325 (17)	0.0294 (15)	0.0297 (18)	−0.0039 (12)	0.0018 (13)	0.0025 (12)
C24	0.0325 (17)	0.0331 (16)	0.0296 (19)	−0.0035 (13)	0.0049 (13)	0.0028 (13)
C25	0.0337 (17)	0.0284 (15)	0.0297 (18)	−0.0010 (12)	0.0062 (13)	0.0047 (12)
C26	0.0362 (19)	0.0362 (17)	0.035 (2)	0.0004 (14)	−0.0013 (14)	0.0015 (14)
C27	0.052 (2)	0.0334 (17)	0.057 (3)	0.0055 (16)	0.0088 (17)	0.0002 (16)
O28	0.0506 (16)	0.0421 (14)	0.0705 (18)	0.0027 (12)	0.0222 (13)	−0.0073 (13)

### Geometric parameters (Å, º)

**Table:**

O1A—C2	1.277 (4)	C15A—H15A	0.95
O1A—H1A	0.84	C16A—C17A	1.383 (5)
C2—C3	1.429 (4)	C16A—H16A	0.95
C2—C11	1.438 (5)	C17A—C18A	1.385 (4)
C3—C4	1.354 (5)	C17A—S1	1.780 (3)
C3—H3	0.95	C18A—C19A	1.389 (4)
C4—C5	1.437 (5)	C18A—H18A	0.95
C4—H4	0.95	C19A—H19A	0.95
C5—C10	1.417 (4)	S1—C20	1.764 (3)
C5—C6	1.420 (5)	C20—C25	1.394 (4)
C6—C7	1.363 (5)	C20—C21	1.401 (4)
C6—H6	0.95	C21—C22	1.389 (4)
C7—C8	1.410 (5)	C21—H21	0.95
C7—H7	0.95	C22—C23	1.385 (5)
C8—C9	1.380 (5)	C22—H22	0.95
C8—H8	0.95	C23—C24	1.410 (4)
C9—C10	1.404 (5)	C23—C26	1.489 (4)
C9—H9	0.95	C24—C25	1.378 (4)
C10—C11	1.453 (4)	C24—H24	0.95
C11—C12	1.404 (4)	C25—H25	0.95
C12—N13A	1.334 (4)	C26—O28	1.232 (4)
C12—H12	0.95	C26—C27	1.500 (4)
N13A—C14A	1.407 (4)	C27—H27A	0.98
C14A—C19A	1.388 (4)	C27—H27B	0.98
C14A—C15A	1.393 (5)	C27—H27C	0.98
C15A—C16A	1.387 (4)		
			
C2—O1A—H1A	109.5	C17A—C16A—C15A	120.2 (3)
O1A—C2—C3	119.1 (3)	C17A—C16A—H16A	119.9
O1A—C2—C11	123.1 (3)	C15A—C16A—H16A	119.9
C3—C2—C11	117.8 (3)	C16A—C17A—C18A	119.1 (3)
C4—C3—C2	122.1 (3)	C16A—C17A—S1	122.1 (2)
C4—C3—H3	118.9	C18A—C17A—S1	118.6 (2)
C2—C3—H3	118.9	C17A—C18A—C19A	121.3 (3)
C3—C4—C5	121.5 (3)	C17A—C18A—H18A	119.3
C3—C4—H4	119.3	C19A—C18A—H18A	119.3
C5—C4—H4	119.3	C18A—C19A—C14A	119.4 (3)
C10—C5—C6	120.1 (3)	C18A—C19A—H19A	120.3
C10—C5—C4	119.3 (3)	C14A—C19A—H19A	120.3
C6—C5—C4	120.6 (3)	C20—S1—C17A	104.88 (15)
C7—C6—C5	121.3 (3)	C25—C20—C21	118.7 (3)
C7—C6—H6	119.4	C25—C20—S1	125.2 (2)
C5—C6—H6	119.4	C21—C20—S1	116.1 (2)
C6—C7—C8	118.7 (3)	C22—C21—C20	120.5 (3)
C6—C7—H7	120.6	C22—C21—H21	119.8
C8—C7—H7	120.6	C20—C21—H21	119.8
C9—C8—C7	121.0 (3)	C23—C22—C21	120.9 (3)
C9—C8—H8	119.5	C23—C22—H22	119.5
C7—C8—H8	119.5	C21—C22—H22	119.5
C8—C9—C10	121.4 (3)	C22—C23—C24	118.3 (3)
C8—C9—H9	119.3	C22—C23—C26	122.6 (3)
C10—C9—H9	119.3	C24—C23—C26	119.0 (3)
C9—C10—C5	117.5 (3)	C25—C24—C23	120.9 (3)
C9—C10—C11	123.6 (3)	C25—C24—H24	119.5
C5—C10—C11	118.9 (3)	C23—C24—H24	119.5
C12—C11—C2	119.0 (3)	C24—C25—C20	120.6 (3)
C12—C11—C10	120.6 (3)	C24—C25—H25	119.7
C2—C11—C10	120.4 (3)	C20—C25—H25	119.7
N13A—C12—C11	122.7 (3)	O28—C26—C23	120.2 (3)
N13A—C12—H12	118.7	O28—C26—C27	120.4 (3)
C11—C12—H12	118.7	C23—C26—C27	119.3 (3)
C12—N13A—C14A	125.6 (3)	C26—C27—H27A	109.5
C19A—C14A—C15A	119.4 (3)	C26—C27—H27B	109.5
C19A—C14A—N13A	123.2 (3)	H27A—C27—H27B	109.5
C15A—C14A—N13A	117.4 (3)	C26—C27—H27C	109.5
C16A—C15A—C14A	120.6 (3)	H27A—C27—H27C	109.5
C16A—C15A—H15A	119.7	H27B—C27—H27C	109.5
C14A—C15A—H15A	119.7		
			
O1A—C2—C3—C4	179.2 (3)	C19A—C14A—C15A—C16A	−0.9 (5)
C11—C2—C3—C4	0.0 (4)	N13A—C14A—C15A—C16A	−179.4 (3)
C2—C3—C4—C5	0.9 (5)	C14A—C15A—C16A—C17A	2.0 (5)
C3—C4—C5—C10	−0.9 (5)	C15A—C16A—C17A—C18A	−1.9 (5)
C3—C4—C5—C6	178.8 (3)	C15A—C16A—C17A—S1	−176.4 (2)
C10—C5—C6—C7	0.6 (5)	C16A—C17A—C18A—C19A	0.8 (5)
C4—C5—C6—C7	−179.2 (3)	S1—C17A—C18A—C19A	175.5 (2)
C5—C6—C7—C8	−0.3 (5)	C17A—C18A—C19A—C14A	0.3 (5)
C6—C7—C8—C9	−0.1 (5)	C15A—C14A—C19A—C18A	−0.2 (5)
C7—C8—C9—C10	0.2 (5)	N13A—C14A—C19A—C18A	178.1 (3)
C8—C9—C10—C5	0.0 (5)	C16A—C17A—S1—C20	−76.5 (3)
C8—C9—C10—C11	179.2 (3)	C18A—C17A—S1—C20	109.0 (3)
C6—C5—C10—C9	−0.4 (4)	C17A—S1—C20—C25	3.5 (3)
C4—C5—C10—C9	179.4 (3)	C17A—S1—C20—C21	−177.5 (2)
C6—C5—C10—C11	−179.7 (3)	C25—C20—C21—C22	0.4 (4)
C4—C5—C10—C11	0.1 (4)	S1—C20—C21—C22	−178.6 (3)
O1A—C2—C11—C12	1.6 (4)	C20—C21—C22—C23	0.7 (5)
C3—C2—C11—C12	−179.3 (3)	C21—C22—C23—C24	−1.1 (4)
O1A—C2—C11—C10	−180.0 (3)	C21—C22—C23—C26	178.0 (3)
C3—C2—C11—C10	−0.8 (4)	C22—C23—C24—C25	0.5 (4)
C9—C10—C11—C12	0.0 (5)	C26—C23—C24—C25	−178.7 (3)
C5—C10—C11—C12	179.2 (3)	C23—C24—C25—C20	0.6 (4)
C9—C10—C11—C2	−178.5 (3)	C21—C20—C25—C24	−1.1 (4)
C5—C10—C11—C2	0.8 (4)	S1—C20—C25—C24	177.9 (2)
C2—C11—C12—N13A	−0.1 (4)	C22—C23—C26—O28	−172.1 (3)
C10—C11—C12—N13A	−178.5 (3)	C24—C23—C26—O28	7.0 (4)
C11—C12—N13A—C14A	−180.0 (3)	C22—C23—C26—C27	7.5 (5)
C12—N13A—C14A—C19A	−0.8 (5)	C24—C23—C26—C27	−173.4 (3)
C12—N13A—C14A—C15A	177.5 (3)		

### Hydrogen-bond geometry (Å, º)

**Table:**

*D*—H···*A*	*D*—H	H···*A*	*D*···*A*	*D*—H···*A*
O1*A*—H1*A*···N13*A*	0.84	1.80	2.558 (4)	149
N13*B*—H13*B*···O1*B*	0.88	1.85	2.558 (4)	136
C9—H9···O28^i^	0.95	2.46 (1)	3.398 (4)	168
C19*A*—H19*A*···O28^i^	0.95	2.56 (1)	3.506 (4)	174
C22—H22···O1*A*^ii^	0.95	2.44 (1)	3.337 (4)	157
C27—H27*B*···O1*A*^i^	0.98	2.49 (1)	3.442 (4)	164

Symmetry codes: (i) *x*−1/2, −*y*+3/2, *z*−1/2; (ii) *x*−1/2, −*y*+3/2, *z*+1/2.
